# Comparison of two non-bronchoscopic methods for evaluating inflammation in patients with acute hypoxaemic respiratory failure

**DOI:** 10.1186/cc7995

**Published:** 2009-08-11

**Authors:** Giuseppe Colucci, Guido Domenighetti, Roberto Della Bruna, Josè Bonilla, Costanzo Limoni, Michael A Matthay, Thomas R Martin

**Affiliations:** 1 Multidisciplinary Intensive Care Unit, Regional Hospital EOC, Via Ospedale 14, Locarno 6600, Switzerland; 2 EOLAB, Ente Ospedaliero Cantonale, Viale Officina 3, Bellinzona 6500, Switzerland; 3 Cantonal Pathological Institute, Lab for Clinical Cytology, Via A. Franzoni 45, Locarno 6600, Switzerland; 4 Department for Social Sciences, University of Applied Sciences and Arts of Southern Switzerland, Le Gerre, Manno 6928, Switzerland; 5 Departments of Medicine and Anaesthesia, Cardiovascular Research Institute, University of California, San Francisco, 505 Parnassus Ave, M917, Box 0624, San Francisco, CA 94143, USA; 6 Medical Research Service of the VA Puget Sound Medical Center and the Division of Pulmonary and Critical Care Medicine, Department of Medicine, University of Washington, Seattle, 1660 S. Columbian Way, Seattle, WA 98108, USA

## Abstract

**Introduction:**

The simple bedside method for sampling undiluted distal pulmonary edema fluid through a normal suction catheter (s-Cath) has been experimentally and clinically validated. However, there are no data comparing non-bronchoscopic bronchoalveolar lavage (mini-BAL) and s-Cath for assessing lung inflammation in acute hypoxaemic respiratory failure. We designed a prospective study in two groups of patients, those with acute lung injury (ALI)/acute respiratory distress syndrome (ARDS) and those with acute cardiogenic lung edema (ACLE), designed to investigate the clinical feasibility of these techniques and to evaluate inflammation in both groups using undiluted sampling obtained by s-Cath. To test the interchangeability of the two methods in the same patient for studying the inflammation response, we further compared mini-BAL and s-Cath for agreement of protein concentration and percentage of polymorphonuclear cells (PMNs).

**Methods:**

Mini-BAL and s-Cath sampling was assessed in 30 mechanically ventilated patients, 21 with ALI/ARDS and 9 with ACLE. To analyse agreement between the two sampling techniques, we considered only simultaneously collected mini-BAL and s-Cath paired samples. The protein concentration and polymorphonuclear cell (PMN) count comparisons were performed using undiluted sampling. Bland-Altman plots were used for assessing the mean bias and the limits of agreement between the two sampling techniques; comparison between groups was performed by using the non-parametric Mann-Whitney-U test; continuous variables were compared by using the Student t-test, Wilcoxon signed rank test, analysis of variance or Student-Newman-Keuls test; and categorical variables were compared by using chi-square analysis or Fisher exact test.

**Results:**

Using protein content and PMN percentage as parameters, we identified substantial variations between the two sampling techniques. When the protein concentration in the lung was high, the s-Cath was a more sensitive method; by contrast, as inflammation increased, both methods provided similar estimates of neutrophil percentages in the lung. The patients with ACLE showed an increased PMN count, suggesting that hydrostatic lung edema can be associated with a concomitant inflammatory process.

**Conclusions:**

There are significant differences between the s-Cath and mini-BAL sampling techniques, indicating that these procedures cannot be used interchangeably for studying the lung inflammatory response in patients with acute hypoxaemic lung injury.

## Introduction

In patients with acute hypoxaemic respiratory failure, acute respiratory distress syndrome (ARDS) represents the more severe form of acute lung injury (ALI) [1]. Although a wide spectrum of clinical disorders may be associated with the development of ALI/ARDS, aetiologies can be divided into diseases associated with direct lung injury (i.e., pneumonia, aspiration, inhalation injury; primary ARDS) and indirect lung injury in the setting of a systemic process (i.e., sepsis, severe trauma with shock, pancreatitis; secondary ARDS) [2]. The inflammatory response of the lung is intense in the alveolar space, and the hallmark of ALI/ARDS in the early phase is severe damage of the alveolocapillary barrier, leading to increased permeability, development of protein-rich and biomarker-rich oedema fluid, and impaired clearance of the oedema [3-5]. The study of the composition and resolution of oedema fluid is of primary importance because it may lead to new insights into the pathogenesis of ALI/ARDS. Sequential sampling of oedema fluid is required for this purpose.

Another common cause of acute respiratory failure is acute cardiogenic lung oedema (ACLE). Although the mechanism of cardiogenic oedema is different from that of ALI/ARDS, recent studies have found that endothelial-derived and epithelial-derived inflammatory mediators are released into the blood even during this form of hydrostatic oedema [6].

Sampling of pulmonary oedema fluid from the distal air spaces is an important procedure that allows the study of the lung inflammatory response. The gold standard technique for this purpose is bronchoscopic bronchoalveolar lavage (bBAL). However, bBAL performed with the standard adult bronchoscope may be poorly tolerated in some critically ill ARDS patients, because it can lead to a worsening of hypoxaemia and hypercapnia, haemodynamic instability, temporary loss of recruited lung areas and development of positive end-expiratory pressure (PEEP) of unknown magnitude [7].

Less invasive bedside techniques have been developed that overcome these difficulties and simplify the procedure, providing alternatives for the rapid study of alveolar fluid in patients with ALI/ARDS. Non-bronchoscopic bronchoalveolar lavage (mini-BAL) and the distal collection of oedema fluid through a simple suction catheter (s-Cath) are examples of these less invasive techniques [4,8,9].

The simple bedside method for sampling distal pulmonary oedema fluid through an s-Cath has been experimentally validated and used in many studies [10]. However, an assessment of inflammation using undiluted sampling obtained by s-Cath in patients with ALI/ARDS and ACLE or a comparison of mini-BAL with s-Cath have not been performed. We therefore designed a prospective study in two groups of patients with acute hypoxaemic respiratory failure, those with ALI/ARDS and those with ACLE, in order to investigate the clinical feasibility of these techniques. To determine whether the two methods can be used interchangeably for sampling the distal air spaces of the lung, we compared mini-BAL and s-Cath for agreement of protein concentration and percentage of polymorphonuclear cells (PMNs), as surrogate markers of acute lung inflammation.

## Materials and methods

**Figure 1:**
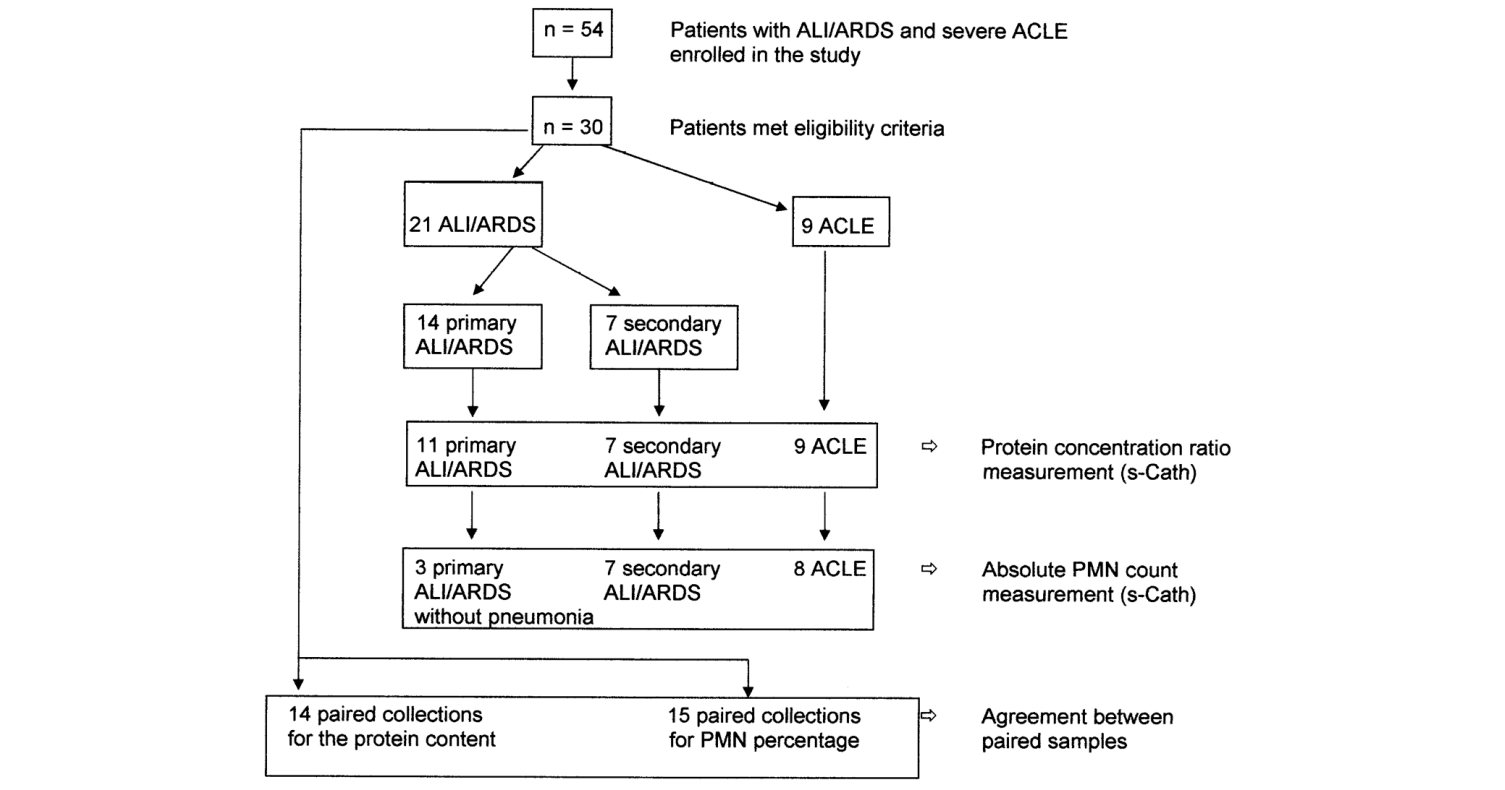
Flowchart of compared subgroups of patients.  ACLE, acute cardiogenic lung oedema; ALI = acute lung injury; ARDS = acute respiratory distress syndrome; PMN = polymorphonuclear cell; s-Cath = suction catheter.

All patients admitted to the multidisciplinary intensive care unit (ICU) of the EOC Regional Hospital "La Carità" in Locarno, Switzerland, between 2002 and 2004 were screened for eligibility. Patients with ALI or ARDS of different causes and with ACLE requiring immediate intubation and mechanical ventilatory support were enrolled (n = 54; Figure 1). Patients with ALI/ARDS were identified by the American-European Consensus Conference definitions [1]. The clinical diagnosis of ACLE was confirmed by reviewing patient records and chest radiographs, recent medical history, and echocardiography or pulmonary artery catheter if the diagnosis was not clear. ACLE was classified as acute exacerbation of congestive heart failure, acute coronary syndrome or exacerbation of diastolic left ventricular dysfunction. Patients were excluded if they had known HIV infection, immunodeficiency necessitating granulocyte colony stimulating factor, ALI/ARDS after thoracic surgery, and mixed causes of pulmonary oedema with elements of both ALI/ARDS and elevated hydrostatic pressure (n = 24). Finally, 30 mechanically ventilated patients met the eligibility criteria (Figure 1). Patients were intubated with oral endotracheal tubes with an internal diameter of 8 mm or more.

This study was approved by the Committee of Human Research of the Canton of Ticino, Switzerland, and informed written consent was obtained from each patient's next of kin.

### Clinical data

Clinical, physiological and biological data at the time of fluid sampling and throughout the hospital course were recorded using a standardised data collection form. The Simplified Acute Physiology Score II (SAPS II) [11] and the Lung Injury Score (LIS) [12] were calculated. Outcome variables included ICU and hospital mortality and length of stay (LOS) in the ICU.

Stable patients were sedated at the time of fluid sampling with midazolam and/or propofol. Ventilatory support in patients with ALI/ARDS was carried out in accordance with the ARDS Network criteria for a protective lung strategy [13]. Patients with ACLE were ventilated with a plateau pressure (P_plat_) limit of 30 cmH_2_O and a pressure-controlled or volume-controlled mode. During sampling with the mini-BAL catheter, arterial oxygen saturation (SpO_2_), haemodynamics (heart rate (HR), systemic arterial pressure (SAP)) and ventilatory variables (expiratory tidal volume (V_t_), minute volume (V_E_), auto PEEP, peak pressure (P_peak_), and P_plat_) were recorded. At the time of fluid collection, patients were treated with vasoactive agents, diuretics, antiarrhythmic agents, antibiotics and fluids (mainly sodium chloride 0.9%). None of the patients were treated with inhaled beta-adrenergic agonists before the sampling procedures.

### Collection of samples

In order to enhance the comparison of the two methods, lung samples with s-Cath and mini-BAL were obtained early in the course of ALI/ARDS and ACLE (within one hour of intubation for the s-Cath and four hours for the mini-BAL).

#### s-Cath

Samples of distal pulmonary oedema fluid were collected without saline instillation by two of the authors (GC, GD) or by trained ICU nurses, following the method previously described by Matthay and co-workers [4,5].

A 14-French (Fr) gauge tracheal s-Cath was blindly advanced through the silicone rubber diaphragm of the swivel adapter of the endotracheal tube into a wedge position in a distal bronchus. Undiluted fluid was then aspirated into a suction trap by gentle suction and stored for less than four hours at 4°C before processing. If the sample was sticky from airway mucus, a small amount (0.2 ml) of sodium citrate was added. The resulting new dilution factor was taken into consideration for the protein content measurements. The collection procedure lasted less than two minutes and was performed without complications in all patients. No modification of ventilatory settings was necessary during the s-Cath procedure.

#### mini-BAL

Mini-BAL was performed by means of a 16-Fr 5 mm outer diameter catheter introduced through a swivel adapter to allow maintenance of PEEP and to set V_E _(BAL Cath, Ballard Medical Products, Draper, UT, USA). By means of the external oxygen port, which allows the catheter to be directed, the 12-Fr inner catheter was advanced until a slight resistance was felt, indicating a wedged position. In three patients, the correct peripheral position of the tip was confirmed by fluoroscopy.

Lavage was performed with 30 ml aliquots of sterile saline, with the goal of instilling a total of 150 ml in five separate aliquots. After each aliquot, a gentle manual suction was applied to recover the instilled fluid. Fluid was kept in specimen traps and immediately processed in the laboratory. Dwell time was as short as possible and the whole procedure lasted less than 15 minutes after the instillation of the first aliquot. The patient's stability was monitored during this procedure by recording SpO_2_, HR, SAP, V_t_, V_E_, auto-PEEP, P_peak _and P_plat_. Arterial blood gas analysis was performed before and 30 minutes after the mini-BAL procedure.

Patients were pre-oxygenated with 100% fraction of inspired oxygen (FiO_2_) 15 minutes prior to sampling. This oxygen concentration was maintained during the sampling collection and for up to 30 minutes after removing the catheter. Then, if SpO_2 _was stable, the pre-BAL FiO_2 _was progressively restored over 30 to 60 minutes. The small 5 mm outer mini-BAL catheter diameter made it possible to maintain the pre-procedure ventilatory settings in most patients during the entire sampling collection [7]; the maintenance of the settings enabled analysis of ventilatory variables (pressures, blood gas) during and after the procedure. A peripheral blood specimen was collected from each patient at the time of the mini-BAL procedure. The mini-BAL procedure was not performed in eight patients because of haemoptysis, major cardiovascular instability or extreme hypoxaemia (partial pressure of oxygen in arterial blood (PaO_2_)/FiO_2 _< 100 with 100% oxygen). During the mini-BAL procedure, 5 of 22 patients experienced minor bronchial bleeding and the procedure was stopped prematurely.

### Measurements

Oedema fluid obtained by means of the s-Cath was filtered through a 100 μm nylon cell strainer (Falcon 2360, Becton Dickinson, Frankling Lakes, NJ, USA). One aliquot (200 μl) was used for cell count (white blood cells (WBCs) and red blood cells (RBCs) respectively, including cell differential), with a Sysmex NE 1500 and the Sysmex K 1000 hematocytometer (Sysmex Europe GmbH, Norderstedt, Germany). Total protein concentration was measured after centrifugation by the Biuret technique. After recording the total volume of mini-BAL fluid, we filtered it through a 100 μm nylon cell strainer; at least 15 ml of the filtered solution was used for measurement of total and differential leukocyte counts. Cell count (WBC, RBC) was performed with a Sysmex NE 1500 and a Sysmex K 1000 hematocytometer. A centrifuged portion of mini-BAL fluid was used for measurement of total protein (Biuret method). The protein content was computed, after centrifugation, by taking into account the total BAL fluid volume for a given patient. The same strategy was used for all patients. The plasma total protein concentration was measured in duplicate by the Biuret method. A protein concentration ratio of oedema fluid:plasma was calculated.

### Statistical analysis

Data are reported as means ± standard deviation or as medians and ranges. Comparison between groups was performed using the non-parametric Mann-Whitney-U test; normally distributed variables were compared by using the unpaired Student *t*-test. Continuous variables (variations of respiratory and haemodynamic variables during mini-BAL) were compared by using Student *t*-test, Wilcoxon signed rank test, analysis of variance or Student-Newman-Keuls test. Categorical variables were compared by using chi-squared analysis or Fisher's exact test. Finally, Bland-Altman plots [14] were used for assessing the mean bias and the limits of agreement between the two sampling techniques, using protein content and neutrophil percentage.

## Results

### Patient characteristics

There were 30 mechanically ventilated patients; 21 with ALI/ARDS (5 with ALI and 16 with ARDS) and 9 with ACLE were studied. The clinical disorders associated with the development of primary ALI/ARDS (n = 14) were pneumonia (n = 11), carmustine-induced lung injury (n = 1), methotrexate-induced lung injury (n = 1) and cryptogenic organising pneumonia (n = 1). Secondary (indirect pulmonary) ALI/ARDS (n = 7) was caused by sepsis (n = 6) and necrotising pancreatitis (n = 1). ACLE was associated with acute coronary syndrome (n = 6), exacerbation of congestive heart failure (n = 2) or left ventricular diastolic dysfunction (n = 1). Patients with ACLE were older than patients with ALI/ARDS and had similarly high SAPS II and LIS. Both groups (ALI/ARDS and ACLE) had a similar impairment in oxygenation (PaO_2_/FiO_2_) at admission and at inclusion in the study. LOS in the ICU was significantly shorter for patients with ACLE. The ICU and hospital mortality rates were lower than expected for patients with ALI/ARDS (19% and 24%, respectively) [15]. In patients with ACLE, the ICU mortality rate was 22%. A summary of demographic and clinical data is shown in Tables 1 and 2.

**Table 1:** Characteristics of patients with ALI/ARDS and ACLE

Variable	ALI/ARDS	ACLE	*P*
Number	21	9	
Age, years	58 ± 18	77 ± 9	0.01
Men/women	16/5	4/5	
PaO_2_/FiO_2 _at intubation	135 ± 69	133 ± 55	0.96
PaO_2_/FiO_2 _at inclusion	160 ± 62	153 ± 49	0.73
CRP at inclusion, mg/L	183 ± 142	79 ± 72	0.05
LIS	2.4 ± 0.5	2.25 ± 0.5	0.42
SAPS II	51 ± 19	66 ± 21	0.06
LOS in ICU, days	14 (2 to 42)^a^	7 (1 to 14)^a^	0.001
ICU mortality (%)	19	22	1.0
Hospital mortality (%)	24	44	0.4

Data shown as mean ± standard deviation.

^a ^Data as median (range)

ACLE = acute cardiogenic lung oedema; ALI = acute lung injury; ARDS = acute respiratory distress syndrome; CRP = C-reactive protein; FiO_2 _= fraction of inspired oxygen; ICU = intensive care unit; LIS = Lung Injury Score; LOS = length of stay; PaO_2 _= partial pressure of oxygen in arterial blood; SAPS II = Simplified Acute Physiology Score II.

**Table 2:** Causes for ALI/ARDS and ACLE

Definition	N
➢ Primary (direct pulmonary) ALI/ARDS	14
• Pneumonia/aspiration	11
• Carmustine-induced lung injury	1
• Methotrexate-induced lung injury	1
• COP	1
➢ Secondary (indirect pulmonary) ALI/ARDS	7
• Sepsis	6
• Necrotising pancreatitis	1
➢ ACLE	9
• Acute exacerbation of CHF	2
• Acute coronary syndrome	
- AMI	5
- Unstable angina	1
• Acute exacerbation of LV diastolic dysfunction	1

ACLE = acute cardiogenic lung edema; ALI = acute lung injury; AMI = acute myocardial infarction; ARDS = acute respiratory distress syndrome; CHF = congestive heart failure; COP = cryptogenic organising pneumonia; LV = left ventricular.

### Variations of haemodynamic and respiratory variables during s-Cath and mini-BAL

s-Cath was performed in all included patients (n = 30) and did not induce changes in haemodynamics or ventilation during or after the procedure.

Mini-BAL was performed in 22 patients (8 patients with ACLE and 14 with ALI/ARDS). The mean value of injected volume was 120 ± 18 ml (range 100 to 150 ml) and the mean recovered volume was 41 ± 15 ml (range 20 to 65 ml). Common haemodynamic variables (HR, SAP) recorded during and 30 minutes after mini-BAL sampling collection were not significantly different from baseline (pre-procedure) in the whole group. By contrast, with an FiO_2 _of 1.0, the SpO_2 _decreased in the whole group from 95 ± 3% at baseline to 93 ± 4% at the end of the procedure (*P *< 0.01) and the PaO_2_/FiO_2 _decreased from 206 ± 68 to 185 ± 51 (*P *= 0.04). The recorded ventilator P_peak _was 28 ± 5 cmH_2_O before and 32 ± 9 cmH_2_O during the procedure (*P *< 0.05); at the end of sampling collection, this pressure returned to the pre-procedure values (28 ± 6 cmH_2_O; *P *< 0.05). The mean V_t _(measured on three consecutive breathing cycles) was 433 ± 41 ml before and 389 ± 43 ml (*P *= 0.50) during sampling.

### Protein concentration ratio, C-reactive protein and PMN count in patients with ALI/ARDS and ACLE

The protein concentration in undiluted oedema fluid sampling obtained by s-Cath was measured in 18 patients with ALI/ARDS (11 primary and 7 secondary ALI/ARDS forms). Three patients with ALI/ARDS were excluded from this analysis because of the presence of thick secretions. The s-Cath procedure allowed us to obtain oedema fluid in all patients with ACLE (n = 9). Comparisons of the protein concentration ratio of oedema fluid:plasma were performed between these groups. The PMN count comparison was performed in 10 patients with ALI/ARDS without pneumonia and in 8 patients with ACLE by using non-contaminated (by airways secretion) undiluted sampling obtained by s-Cath. The PMN count was not possible because of thick secretions in eight patients with ALI/ARDS and because of an insufficient quantity of oedema fluid in one patient with ACLE. For the Bland-Altman analysis of agreement between the two sampling techniques, with protein content and neutrophil percentage as parameters, we used only simultaneously collected mini-BAL and s-Cath paired samples. Paired collection was possible in 14 patients for the protein content (8 patients with ALI/ARDS without thick secretions and 6 patients with ACLE) and in 15 patients for PMN percentage determination (9 patients with ALI/ARDS without thick secretions and 6 patients with ACLE; Figure 1).

**Figure 2:**
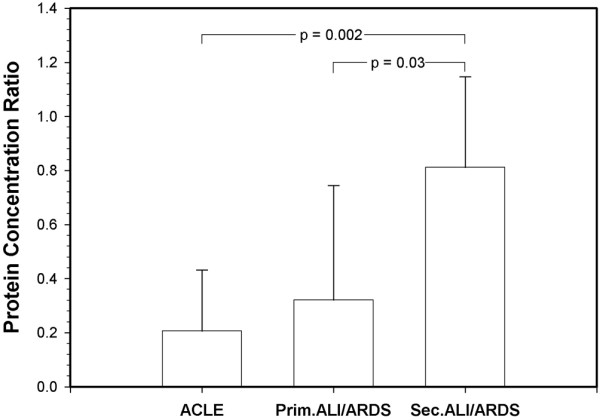
Protein concentration ratio in patients with ACLE (n = 9), primary (n = 11) and secondary (n = 7) ALI/ARDS.  Sampling obtained by s-Cath. ACLE = acute cardiogenic lung oedema; ALI = acute lung injury; ARDS = acute respiratory distress syndrome; s-Cath = suction catheter.

**Figure 3:**
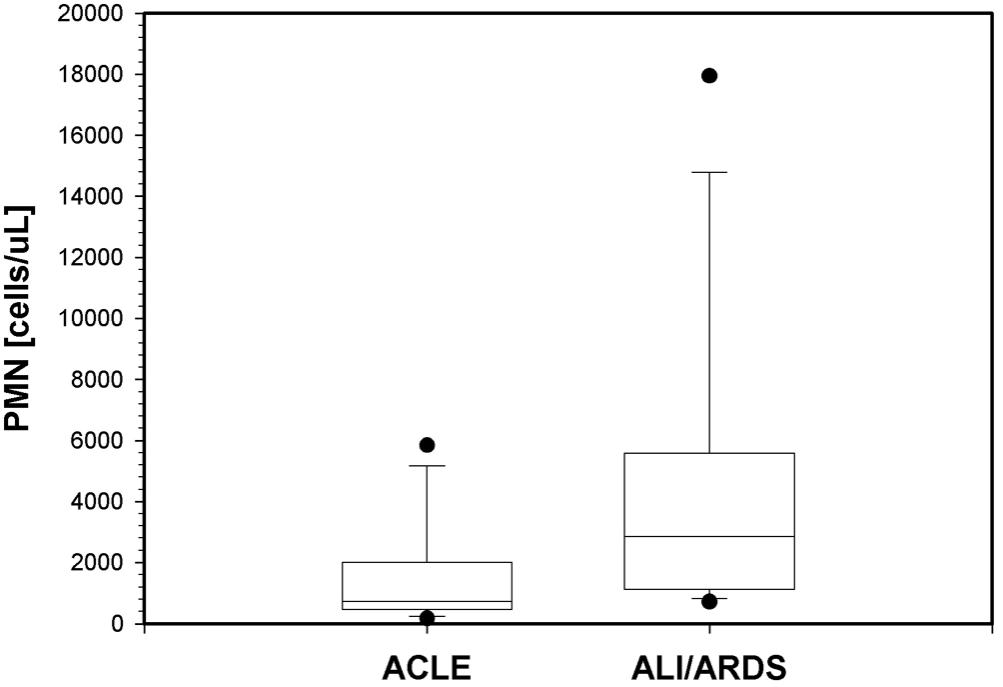
Absolute PMN count in patients with ACLE (n = 8) and ALI/ARDS without pneumonia (n = 10).  The horizontal line represents the median. The box encompasses the 25^th ^to 75^th ^percentiles and the error bars show the 10^th ^to 90^th ^percentiles. Filled circles: outliers. The difference is non-significant. Sampling obtained by s-Cath. ACLE = acute cardiogenic lung oedema; ALI = acute lung injury; ARDS = acute respiratory distress syndrome; PMN = polymorphonuclear cell; s-Cath = suction catheter.

As shown in Figure 2, the mean ratio of oedema fluid (obtained by s-Cath) to plasma protein in patients with ACLE (n = 9) at the time of intubation was 0.20 ± 0.19, a value significantly different from that found in patients with ALI/ARDS with a secondary (indirect) origin (n = 7; 0.81 ± 0.33; *P *= 0.002). Patients with primary ALI/ARDS (direct pulmonary, mainly pneumonia; n = 11) had a mean ratio value of 0.32 ± 0.42 (*P *= 0.03 vs. secondary ALI/ARDS protein concentration ratio). The mean plasma C-reactive protein level at inclusion was 183 ± 142 mg/L in the whole ALI/ARDS group (n = 21) and 79 ± 72 mg/L in patients with ACLE (n = 9; *P *= 0.05; Table 1). Figure 3 shows the median value of the absolute PMN count for all but one of the patients with ACLE (n = 8) compared with the PMN count for patients with ALI/ARDS without pneumonia (n = 10), obtained by s-Cath. There was no statistically significant difference between groups. The patients with ACLE also showed an increased PMN count, but this was not as great as that observed in the patients with ALI/ARDS.

### Evaluation of agreement between s-Cath and mini-BAL sampling methods

**Figure 4:**
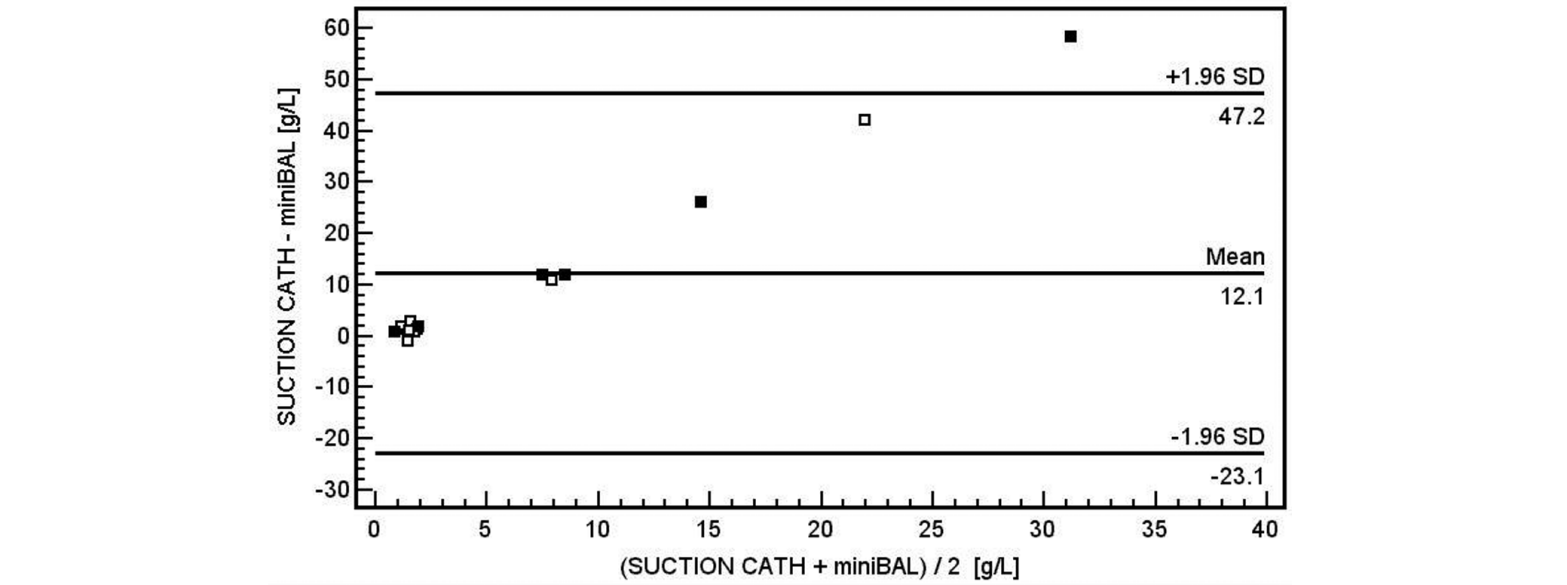
Bland-Altman analysis of agreement showing the differences between protein content (g/L) measurements plotted against the average between methods. Squares correspond to patients. The middle horizontal line indicates the average difference between the two methods (12.1 g/L), whereas the outer lines represent the upper and lower limits of agreement. The black squares represents patients with acute cardiogenic lung oedema.

**Figure 5:**
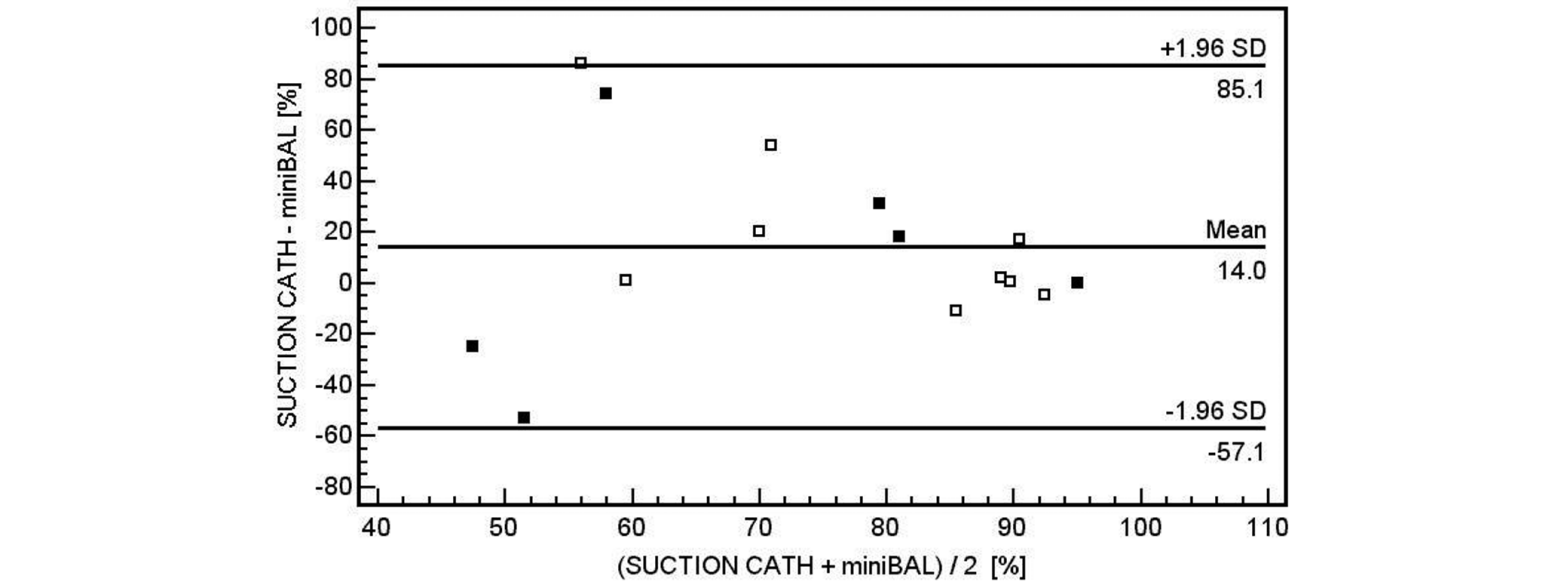
Bland-Altman analysis of agreement showing the differences between measurements of percentage of neutrophils plotted against the average between methods.  Squares correspond to patients. The middle horizontal line indicates the average difference between the two methods (14%), whereas the outer lines represent the upper and lower limits of agreement. The black squares represents patients with acute cardiogenic lung oedema.

Bland-Altman plots evaluating agreement between the two sampling techniques using protein content and PMN percentage as efficacy parameters are shown in Figure 4 and 5. The average difference in protein content was 12.1 g/L (n = 14 paired collections, 6 patients with ACLE and 8 patients with ALI/ARDS without thick secretions; *P *= 0.025; 95% confidence interval (CI) 1.73 to 22.4), indicating that the protein content detected in the same patient was significantly higher when sampled by s-Cath. The differences increase as the average protein content increases in the two methods (Figure 4). Specifically, as the average total protein concentration in the lung increases, the s-Cath method returns more protein than does the mini-BAL method. The average difference in the PMN percentage was 14.0% (n = 15 paired collections, 6 patients with ACLE and 9 patients with ALI/ARDS without thick secretions; *P *= 0.16; 95% CI -6.12 to 34.05), indicating that the PMN percentage detected by the two techniques in the same patient was not significantly different. The power of this test was nevertheless only 65% with our paired sample size of 15 patients. The difference between the two techniques tended to decrease as the average PMN percentage increased (Figure 5). Finally, we did not find any association related to the underlying disease process.

## Discussion

The sampling of alveolar fluid from patients with acute respiratory failure allows the study of lung inflammatory response to various injuries. As demonstrated by Matthay and co-workers [4,5,10], direct sampling of undiluted lung oedema fluid may provide fundamental insights into the onset and evolution of acute inflammatory changes in permeability lung oedema as well as help to determine whether the pulmonary oedema is primarily from a hydrostatic or increased permeability mechanism. bBAL is generally well tolerated even in severely hypoxaemic patients with ALI/ARDS [15]. Yet, the sampling collections with this gold standard technique may sometimes be restricted by persistent severe hypoxaemia or cardiovascular instability, the presence of small endotracheal tubes or the unavailability of a bronchoscopist. Mini-BAL has been successfully evaluated in comparison with bBAL for the diagnosis of ventilator-associated pneumonia [8], but it showed disappointing results in a recent study where both techniques were compared for assessing alveolar permeability and inflammation in patients at risk for ARDS or with ARDS [9]. However, considering that the s-Cath sampling might not perform adequately after 24 hours because the ability to obtain oedema fluid may decline over the course of ALI/ARDS, we decided to use the mini-BAL as a comparison methodology, because it is easily performed at the bedside and may be completed without a bronchoscopist.

Mini-BAL was generally a safe procedure. However, when performed with a 16-Fr 5 mm outer diameter catheter, the mini-BAL procedure on rare occasions induced significant gas exchange abnormalities lasting up to 30 minutes after the end of collection, as shown by SpO_2 _and PaO_2_/FiO_2_abnormalities. Hypoxaemia was probably induced by the lavage itself and by reduced tidal volumes delivered while the catheter was in place [7,16]. We measured the P_peak _on the ventilator (back pressure) before, during and after the procedure. This pressure significantly increased during mini-BAL sampling, representing an indirect sign of unstable tidal volumes during the ventilatory cycle [7,17]. Moreover, mini-BAL caused minor bronchial haemorrhage in five patients, leading us to stop the investigation prematurely. Another potential side effect of mini-BAL is sepsis-like systemic effects, which may emerge predominantly in patients with pneumonia [18]. With s-Cath, samples were collected by physicians and trained ICU nurses; the procedure was rapid and no complications occurred. This result indicates an advantage of the s-Cath procedure because collection can be performed shortly after intubation and at the onset of ALI or hydrostatic oedema. Another advantage is that fluid is suctioned undiluted without saline, and, therefore, the measurement of protein or potential mediators of lung injury can be made without dilution. For this reason, the protein concentration ratio of the oedema fluid:plasma was calculated in our different groups using samples obtained by the s-Cath. The main disadvantage of the s-Cath oedema fluid sampling method is that it seldom yields lung oedema fluid after the first 24 hours of intubation. Therefore, this sampling technique is preferred for studying lung fluid at the onset of lung injury shortly after endotracheal intubation.

In the patient population in this study, the mean value of the oedema fluid protein/plasma ratio in patients with primary ALI/ARDS was significantly lower compared with the value in the group of patients with a secondary form of ALI/ARDS. We speculate that during secondary ARDS, there is a more severe capillary leak that may flood the alveoli [19], possibly explaining the higher protein concentration ratio in the early disease phase of indirect ALI/ARDS while the early direct insult of the alveoli in pneumonia-associated ALI/ARDS may exude less protein resulting in a lower oedema fluid/plasma ratio.

Our results did not show a good agreement between the s-Cath and mini-BAL sampling techniques. Using protein content and PMN percentage as efficacy parameters, we found, in applying Bland-Altman plots, a significant bias with wide limits of agreement between the two methods. When the protein concentration in the lung was high, the s-Cath method is a better method for estimating protein concentration (Figure 4); in contrast, as inflammation increases, both methods provide similar estimates of the percentage of neutrophils in the air spaces of the lung (Figure 5). The analysis of our plots indicates that, compared with the results for mini-BAL, the protein content was significantly higher in the same patient when measured by s-Cath. In other words, the s-Cath sampling technique 'detected more' protein content, meaning that this method could be more sensitive than mini-BAL itself for this purpose. These results suggest that the s-Cath and mini-BAL procedures cannot be used interchangeably for studying lung fluid composition during lung injury and that collection of lung oedema fluid should be performed with the same method.

Interestingly, our results show an increased absolute PMN count recorded in patients with ACLE. Recent laboratory and clinical studies have provided evidence that cardiogenic pulmonary oedema may be associated with a mild increase in permeability of the alveolocapillary barrier and that ongoing pulmonary injury and inflammation may characterise this disorder, particularly when the hydrostatic pressure elevations are severe [20-24]. For example, Pugin and colleagues [25] found that inflammatory cytokines and IL-8 increased rapidly after intubation and positive pressure ventilation in patients with ACLE, although these levels were lower than in patients with ALI. Considering that our samples were obtained shortly after intubation through the s-Cath procedure, the increased absolute PMN count in patients with ACLE was probably not related to ventilator-induced lung injury. We speculate that this finding may indicate an inflammatory process during the hydrostatic form of pulmonary oedema. Although the mean plasma C-reactive protein level in patients with ACLE was significantly lower than the level recorded in the group of patients with ALI/ARDS, the raised C-reactive protein concentration in patients with the hydrostatic form of lung oedema, devoid of any treatment with corticosteroids or clinical and bacteriological evidence of infection, is notable. Dysregulation of C-reactive protein in the setting of acute hydrostatic lung oedema seems to be a common finding that could be associated with a concomitant inflammatory process, therefore perhaps playing a role in the evolution of this form of oedema [26,27].

Our study has some limitations. A lack of agreement between s-Cath and mini-BAL may occur for several reasons: the variability of instilled volume of the mini-BAL may have influenced the results; the techniques have two distinct dilution features, the region of the lung where oedema is sampled is achieved blindly and the lung injury is heterogeneous; and the difficulty in wedging the mini-BAL catheter properly in a distal airway may further represent a barrier in achieving comparable results. Another limitation for using s-Cath is the presence of sticky airways secretions, typically found in primary ALI/ARDS following bilateral pneumonia, making it impossible to obtain free-flowing oedema fluid. This problem was the main reason for excluding few patients from our paired analysis.

Studies assessing the impact of pulmonary heterogeneity in patients with ALI, ARDS or ACLE would therefore be helpful in the future for evaluating sampling agreement of different techniques. Finally, although we tried to study our patients as early as possible after the clinical recognition of injury, some patients were not investigated with the mini-BAL procedure at exactly the same time as the s-Cath sampling but all the procedures were completed within a four-hour time window. Nevertheless, we consider this frame of time as likely to be representative of the functional status of lung neutrophils and protein concentration because lung PMN and total protein does not change significantly over the first three days after the onset of ARDS when measured by the traditional bBAL procedure [28-30].

## Conclusions

This study in patients with ALI/ARDS and cardiogenic lung oedema compared two minimally invasive methods for sampling oedema fluid from distal lung air spaces. The results show significant differences between the s-Cath and mini-BAL techniques, suggesting that these procedures cannot be used interchangeably for sequentially studying the lung inflammatory response in the distal air spaces. Except for use in patients with purulent airway secretions, the s-Cath method has more advantages than the mini-BAL technique, because the s-Cath procedure is rapid, non-invasive, inexpensive and, above all, can be performed shortly after intubation at the onset of ALI or hydrostatic oedema. Moreover, the oedema fluid is undiluted with saline, allowing the accurate measurement of protein and potential mediators of lung injury. The oedema fluid sampling technique remains a preferred method for studying lung fluid at the onset of ALI in intubated patients. Nevertheless, both techniques are minimally invasive and provide a method to quantify the inflammatory response and the degree of protein exudation in the distal airspaces of the lung in patients with bilateral pulmonary infiltrates and acute respiratory failure that requires mechanical ventilation.

## Key messages

• No data exist comparing mini-BAL and s-Cath for assessing lung inflammation in acute hypoxaemic respiratory failure.

• Using protein content and PMN percentage as parameters, we identified substantial variations between the two sampling techniques.

• When the protein concentration in the lung was high, the s-Cath was a more sensitive method.

• As inflammation increased, both methods provided similar estimates of neutrophil percentages in the lung.

• Both procedures cannot be used interchangeably for sequentially studying the lung inflammatory response in the distal air spaces.

## Abbreviations

ACLE: acute cardiogenic lung oedema; ALI: acute lung injury; ARDS: acute respiratory distress syndrome; bBAL: bronchoscopic bronchoalveolar lavage; CI: confidence interval; FiO_2_: fraction of inspired oxygen; Fr: French; HR: heart rate; ICU: intensive care unit; IL: interleukin; LIS: Lung Injury Score; LOS: length of stay; mini-BAL: non-bronchoscopic bronchoalveolar lavage; PaO_2_: partial pressure of oxygen in arterial blood; PEEP: positive end-expiratory pressure; PMN: polymorphonuclear cell; P_peak_: peak pressure; P_plat_: plateau pressure; RBC: red blood cell; SAP: systemic arterial pressure; SAPS II: Simplified Acute Physiology Score II; s-Cath: suction catheter; SpO_2_: pulsed oxygen saturation; V_E_: minute ventilation; V_t_: expiratory tidal volume; WBC: white blood cell.

## Competing interests

The authors declare that they have no competing interests.

## Authors' contributions

GD collected the samples and wrote the initial draft and the final manuscript. GC collected the samples and data and participated in writing and revising the final manuscript. RDB and JB performed the data analysis. CL directed the statistical analysis and interpretation and participated in drafting the initial manuscript. MAM and TRM conceived the premise and participated in writing, interpretation and analysis. All authors have read and approved the final manuscript.
